# Bridging the digital divide for outpatients treated with anticancer chemotherapy: a retrospective quantitative and qualitative analysis of an adapted electronic Patient Reported Outcome program

**DOI:** 10.1007/s00520-025-09171-9

**Published:** 2025-01-30

**Authors:** Coralie Boiteau, Natividad Alarcon, Charlotte Joly, Charlotte Fenioux, Claire Queval, Sylvie Dutendas, Isabelle Bartoszczyk, Hadjer Ben Nadji, Meriem Bouayed, Claude Ganter, Naël Quatrehomme, Catherine Agius, Christophe Tournigand, Emmanuelle Kempf

**Affiliations:** 1 Department of Medical Oncology, Assistance Publique - Hôpitaux de Paris, Henri Mondor Teaching Hospital, Créteil, France 1 Rue Gustave Eiffel, 94000; 2 MN Santé, Lyon, France; 3 Headquarters, Direction de La Stratégie et de la Transformation, Mission Handicap, Assistance Publique - Hôpitaux de Paris, Paris, France; 4 Paris-Est Créteil University (UPEC), Inserm, U955 Créteil, France; 5 Laboratoire d’Informatique Médicale Et d’Ingénierie Des Connaissances Pour La E-Santé (LIMICS), Sorbonne University, Inserm, Paris, France

**Keywords:** (MeSH terms): Telemedicine, E-Health Services Research, Neoplasms, Quality of health care, Routinely collected health data, Social deprivation

## Abstract

**Purpose:**

Using electronic patient-reported outcomes (ePRO) in clinical trial has shown benefits for patients. However, the digital divide can lead to unequal access to telehealth. We investigated whether a dedicated support program could bridge that divide.

**Methods:**

Between February 2021 and June 2022, outpatients undergoing chemotherapy for cancer at our teaching hospital in France were given the Onco’nect® ePRO application if they were affected by the digital divide. They were also offered a dedicated support program that included the lending of a tablet, access to healthcare professionals, training, technical support, and peer-to-peer guidance. We conducted semi-structured interviews to assess the challenges they faced.

**Results:**

We enrolled 22 patients, of whom 10 (45%) made good use of the application and completed > 50% of the questionnaires in the application, while 5 (23%) completed > 75%. However, 12 (55%) of the 22 patients remained poor users of the application over a median participation of 4 months (IQR, 3–7). We also measured social deprivation but found no association with questionnaire completion rate. The under-use of Onco’nect® was due not only to the patients’ understanding of its clinical benefit or to their computer skills, but also to poor health literacy and strong emotional responses to using the application.

**Conclusion:**

Dedicated support programs help many patients make the most of telehealth. However, most of our patients in the digital divide under-used the ePRO application, primarily due to their poor health literacy.

**Supplementary Information:**

The online version contains supplementary material available at 10.1007/s00520-025-09171-9.

## Background

The management of adverse events (AEs) of systemic cancer treatment is a daily challenge for patients, caregivers, and clinicians [1–3]. Remote monitoring of chemotherapy treatment may help overcome this challenge. It has been shown to improve patient quality of care [4, 5] by strengthening the link between outpatients and healthcare professionals. It has also been found to improve medical support at home by promoting interactive communication and patient reassurance [6–8]. The SARS-CoV-2 pandemic emphasized the benefit of telemedicine in cancer care [9, 10]. Telehealth also referred to as telemedicine by Medicaid involves “two-way, real-time interactive communication between the patient and the physician or practitioner at the distant site. This communication often requires the use of interactive telecommunications equipment” [11]*.* Telemedicine reduces both unscheduled referrals to the emergency room and treatment side effects by speeding up their reporting and management. Telemedicine also improves patient treatment compliance and overall survival [12]. Yet not all patients have access to, or know how to use, telemedicine applications [13–15]. This fact may limit remote monitoring of cancer treatment.

The digital divide refers to the disparity between countries, regions, and people in their access to digital services; this is the gap between populations of different socioeconomic backgrounds with respect to their access to the Internet and communication technologies in general. This gap depends on myriad factors, one of which is, of course, affordability [13, 16]. Data suggest that 66% of the world population had access to the Internet in 2023, but that 2.77 billion people did not [14]. In France, a survey showed that 12% of the population over 15 years of age had no Internet access from home, regardless of the type of connection or device (computer, tablet, mobile phone) [15]. This last study showed that the digital divide may also take another form: digital illiteracy, which is an inability to use computers and the Internet. According to a study by INSEE, the French statistics body, digital illiteracy may have impacted up to 17% of French people in 2019 [17].

Cancer patients can only benefit from telemedicine if they have access to, and knowledge of, digital tools. Empowerment enables patients to take ownership of information about their condition, make informed decisions, and adhere to treatment [18]. A systematic review has suggested that cancer patient support by peers or patient-partners may improve their empowerment [19]. In line with this suggestion, our study included a PP to improve patient empowerment.

In 2015, Irizarry et al. showed that patients’ ability to use digital tools depended on their age, education, and health literacy [20]. The Organisation for Economic Co-operation and Development defines literacy as “the ability to understand and use written information in everyday life, at home, at work and in the community to achieve personal goals and to expand one’s skills and abilities” [21]. The World Health Organization (WHO) defines health literacy as the set of personal characteristics and social resources that individuals and communities need to access, understand, evaluate, and use information and services to make health decisions [22]. For example, low health literacy is often associated with poorer health due to less access to screening and less understanding of medical treatments [23, 24].

Since 2017, all outpatients with solid cancers undergoing intravenous chemotherapy at the Henri Mondor Teaching Hospital, Paris, are offered remote monitoring using Onco’nect®, a tool for capturing and responding to electronic patient-reported outcomes (ePRO) [25]. Two days before and 2 days after cancer treatment administration, patients are sent a standardized digital questionnaire that assesses AEs. The questionnaire is based on the National Cancer Institute’s Patient-Reported Outcomes version of the Common Terminology Criteria for Adverse Events (PRO-CTCAE®) (v1.0). According to a pre-formatted algorithm (Supplemental Table 1), grade > 2 AEs trigger an alert system that sends an email to a nurse navigator [26, 27]. Patients can generate an “I don’t feel good” emergency alert, chat with the nurse navigator, and share digital information such as lab results, pictures, or other documents. Onco’nect® data is stored in a data mart of the MiPih health data hosting service. Onco’nect® received approval from CNIL, the French data protection agency (approval number 1957171, v0) [28].

**Table 1:** Patient characteristics in the overall and interview population

Characteristic	Overall population (*N* = 22)	Patients interviewed (*N* = 8)
**Gender** Female *n* (%)	15 (68)	3 (37)
**Age** (median, IQR) < 50 *n* (%) 50–59 *n* (%) 60–69 *n* (%) 70–79 *n* (%) > 80 *n* (%)	69 (64–74) 1 (5) 4 (18) 6 (27) 9 (41) 2 (9)	69 (62–77) 1 (12.5) 1 (12.5) 2 (25) 3 (37.5) 1 (12.5)
**Marital status** *n* (%)		
Married Divorced Widow Single In a relationship	6 (27) 5 (23) 3 (14) 6 (27) 2 (9)	2 (25) 2 (25) 1 (12.5) 2 (25) 1 (12.5)
**Employment** *n* **(%)**		
Employed Retired Disability Unemployed NR	3 (13) 15 (68) 1 (5) 2 (9) 1(5)	0 (0) 6 (75) 0 (0) 1 (12.5) 1 (%)
**EPICES** score (median, IQR) < 30 *n* (%) ≥ 30 *n* (%) NR *n* (%)	39 (26–50) 5 (22) 13 (60) 4 (18)	31 (14–47) 2 (25%) 5 (62.5%) 1 (12.5)
**Performance status** *n* **(%)**		
0 1 2	8 (36) 9 (41) 5 (23)	4 (50) 3 (37.5) 1 (12.5)
**Cancer stage** *n* (%)		
Localized Locally advanced Metastatic	5 (23) 2 (9) 15 (68)	3 (37.5) 1 (12.5) 4 (50)
**Primary tumor site** *n* (%)		
Breast Gastrointestinal Prostate Urological	3 (14) 14 (63) 3 (14) 2 (9)	1 (12.5) 4 (50) 3 (37.5) 0 (0)
**Prior treatment lines** *n* (%)		
1 2 3	17 (77) 3 (14) 2 (9)	5 (62.5) 2 (25) 1 (12.5)

*EPICES* Evaluation of Precariousness and Health Inequalities in Health Examination Centers, *IQR* interquartile range, *NR* not recorded.

Our study evaluated a dedicated remote monitoring program for patients facing the digital divide and explored the challenges of using such a program. Our study endpoints were (i) the Onco’nect® questionnaire completion rate among patients and (ii) the association between the questionnaire completion rate and patients’ social deprivation.

## Methods

### Patient inclusion and study intervention

Between February 2021 and June 2022, a program was launched to enable cancer outpatients in the digital divide to benefit from Onco’nect® remote monitoring. A patient-partner was involved in the project from the outset and accompanied the patients throughout the study. We included outpatients starting systemic anticancer treatment who were interested in the remote monitoring tool but were in a digital divide setting. In other words, eligible patients had no smartphone, tablet, computer, or home Internet connection, nor were they able to buy or pay for an Internet connection. They had to be able to use a tablet and to speak and read French. Patients were lent a simplified, user-friendly tablet intended for older patients with a 4G connection. Patients were identified after an initial assessment by a registered nurse. They were provided with peer-to-peer support and an educational program. An initial visit with a patient-partner and a research engineer was organized to show them how to use the tablet and Onco’nect® at home, using adapted educational tools created by the patient-partner (Supplemental Fig. 1). If necessary, patients could repeat this initial visit. Several meetings were held between the patient and the cancer supportive care team to assess and address patient difficulties related to the use of Onco’nect® and the tablet. The number of questionnaires varied from patient to patient, as they were not all included for the same length of time. There were 28 questions per questionnaire. Incomplete questionnaires could not be submitted to the care team. Our results were, therefore, based on the number of fully completed questionnaires.

**Fig. 1:**
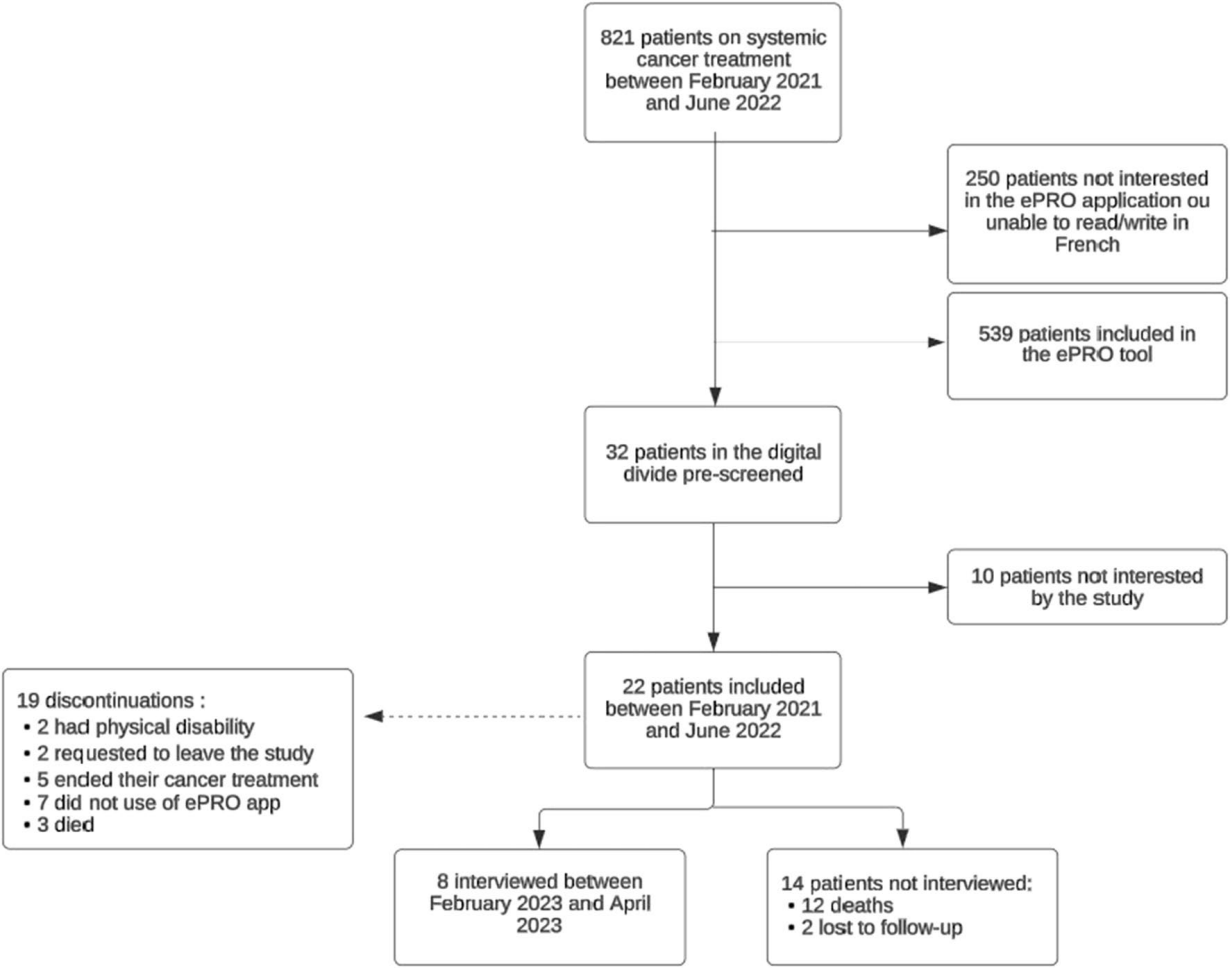
Study flow chart. Abr: ePRO, Electronic Patient Reported Outcomes

### Quantitative analysis

The primary endpoint was the Onco’nect® questionnaire completion rate. Patients were categorized into four categories according to their overall questionnaire completion rate: very good completers (≥ 75% of questionnaires completed), good completers (50–74%), bad completers (26–49%), and very bad completers (≤ 25%). A nurse navigator and a research engineer retrospectively collected the following data from Onco’nect® between January and March 2023: patient gender, age, marital status, employment status, patient performance status at baseline (based on the WHO scale and extracted manually from medical records), stage and type of primary cancer, number of previous treatment lines and chemotherapy cycles, reason for end of inclusion, duration of inclusion, number of meetings with the patient-partner and research engineer, response to questionnaires, number and type of alerts, type of alert management (nurse navigator alone or assisted by a medical oncologist), number of ER and supportive care referrals, and number of chat messages sent. Patient social deprivation was defined as an Evaluation of Precariousness and Health Inequalities in Health Examination Centers (EPICES) score ≥ 30 as assessed by a social worker (score ranges from 0 to 100) [20]*.* We used the box plot to display the distribution of patients according to EPICES score and questionnaire completion rate. We performed a *χ*^2^ test to assess the association between questionnaire completion rate and EPICES score, the significance level being 0.05.

### Qualitative analysis

The secondary endpoints measured patients’ difficulties regarding the use of Onco’nect®, and what improvements they would suggest. The nurse navigator met with all outpatients based on their performance status and readiness to perform an interview. Semi-structured face-to-face and phone-based interviews were conducted by the nurse navigator and recorded between February and April 2023 at the Henri Mondor Hospital during routine follow-up visits. The interview scoring grid was developed with a clinical psychologist specializing in qualitative research. During the interviews, patients were asked about the clinical benefit of Onco’nect®, their understanding of the questionnaire and of the vocabulary used, the relevance of the questions, and their ability to read and write in French. At the end of the interview, “MediPicto” pictograms were presented to the patient, who was asked whether the pictograms would be a useful addition to the Onco’nect® questionnaire. MediPicto is a free application created by the AP-HP university hospital center of which our hospital is a part. Its purpose is to facilitate communication between healthcare professionals and patients who have difficulty expressing or understanding clinical signs and symptoms (Supplemental Fig. 1) [29]. All interview data were analyzed thematically and included a verbatim recording of the patient’s words.

### Institutional approval

Our hospital’s Scientific and Ethics Committee (IRB 00011558) approved this qualitative and quantitative single-center retrospective study on February 23, 2023. The study did not meet French criteria to be deemed human subjects research. Only data that was strictly necessary to the aims of our study were collected and analyzed. This study was conducted in accordance with the Declaration of Helsinki and reported using the STROBE and COREQ checklists for the quantitative and qualitative parts of our study, respectively [30, 31].

## Results

### Patient characteristics

Between February 2021 and June 2022, 821 outpatients received intravenous cancer treatment. Among them, 539 were included in the Onco’nect® ePRO tool. A total of 32 were preselected for the study because they were in the digital divide, of whom 22 were included (Fig. 1). Half of the patients were more than 70 years old, and 13 were socially deprived. Most patients had metastatic gastrointestinal cancer and were in their first line of treatment. Patient demographics and disease characteristics are summarized in Table 1.

### Patient use of Onco’nect®

**Fig. 2:**
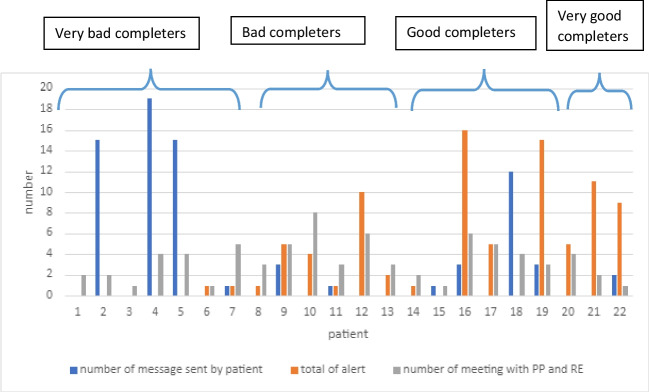
Number of chat messages sent, of alerts triggered and of meetings between the patient and the research engineer (RE) and patient partner (PP), per patient categorized according to the questionnaire completeness

**Fig. 3:**
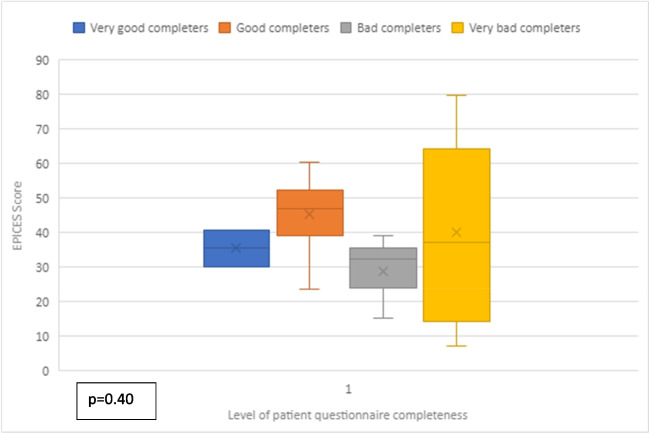
Level of patient social deprivation (EPICES score) according to the completeness of Onco’nect® questionnaires. The patient social deprivation was defined as an Assessment of Precariousness and Health Inequalities in Health Examination Centers (EPICES) score ≥ 30 ranging from 0 to 100. Box plot is based on 19 patient because of missing EPICES score data

**Fig. 4:**
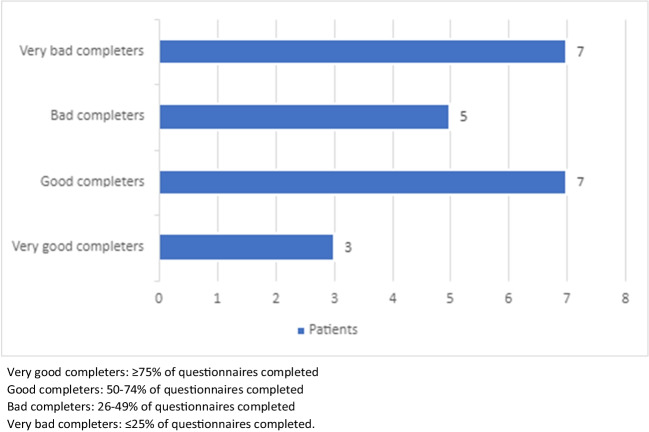
Distribution of patients according to their level of questionnaire completion. Very good completers: ≥ 75% of questionnaires completed. Good completers: 50–74% of questionnaires completed. Bad completers: 26–49% of questionnaires completed. Very bad completers: ≤ 25% of questionnaires completed

The number of questionnaires varied from patient to patient, as they were not all included for the same length of time. There were 28 questions per questionnaire, all of which the patient had to answer to submit the questionnaire. Our results were based, therefore, on the number of fully completed questionnaires. Table 2 summarizes patient use of Onco’nect® over a median of 4 months (IQR 3–7). Figure 4 summarizes the distribution of patients according to their level of questionnaire completion. Some 10 of the 22 patients (45%) were categorized as good or very good completers. Chat messages were mainly sent by patients who did not trigger any alert or complete any questionnaire. Patient 10 had eight meetings with the patient-partner and research engineer and remained a bad completer (40%) (Fig. 2). Figure 3 plots questionnaire completion rate as a function of EPICES social deprivation score. The *p*-value reached 0.4, indicating that there was no statistical relationship. Most good completers were socially deprived.

**Table 2:** Use of Onco’nect® in the overall and interview population

	Overall population (*N* = 22)	Patients interviewed (*N* = 8)
**Patient participation duration (months)** Median (IQR)	4 (3–7)	4 (1–8)
**Number of chemotherapy cycles** Median (IQR)	8 (4–14)	8 (4–13)
**Number of patient meetings with PP/RE** Median (IQR)	3 (2–4)	2 (1–4)
** Number of patients by completion rate n (%)**		
Very bad completers Bad completers Good completers Very good completers	7 (32) 5 (22) 7 (32) 3 (14)	4 (50) 0 (0) 3 (38) 1 (12)
**Number of alerts triggered by patient Median (IQR)**		
Orange Red	3 (1–5) 3 (1–7)	0 (0–0) 0 (0–1)
**Alert management** *n* (%)		
By nurse alone By nurse with oncologist Referral to emergency room Referral to supportive care	80 (84) 15 (16) 3 (3) 2 (2)	16 (94) 1 (6) 0 (0) 1 (6)
**Number of chat messages sent** Median (IQR)	3 (1–13)	1 (0–12)

*IQR* interquartile range, *PP* patient-partner, *RE* research engineer.

### Patients’ subjective experience of Onco’nect®

The patients interviewed (*n* = 8) were mainly male and had a low EPICES score (Table 1). One patient refused to be recorded. Half were very bad completers, triggered few alerts, and sent few messages. All patients met at least once with the patient-partner and research engineer (Table 2). Our analysis of the interviews revealed the following three overarching themes (Table 3 and Supplemental Table 2):

**Table 3:** Feedback on using Onco’nect® as reported by 8 interviewees

Understanding the clinical benefit of remote monitoring	“it’s to check how I’m getting on” “For better care … better support” “To feel safe”
Understanding the wording of the questionnaire	“There may be a word or two that can be hard to understand” “To be honest, I don’t read much” “It would be nice to have images like that” “[images] can help with sentences you don’t understand” “I only speak French, I get confused, I read it very badly, a few words, it’s complicated” “I can’t speak French very well; I don’t read it well either …”
Emotions	“Fear of ticking the wrong box” “Feels like confession” “Sometimes I make spelling mistakes” “It’s a hang-up, maybe; I’m afraid of not doing it properly” “I tried to catch up”

- Understanding the clinical benefit of remote monitoring
  Patients understood the benefits of remote monitoring via Onco’nect® and all of them reported that it was a good idea. Three patients referred to “personal safety.” Two patients did not understand the purpose of the ePRO application: one thought it was “a gift” and the other that it allowed communication between patients.
- Understanding the wording of the questionnaire
  All patients said that they had no difficulty with reading and writing in French. Half of the patients reported that they did not read every day. Two patients reported that they did, but then retracted their statement. None of the patients criticized the content of the questionnaire directly. Six patients thought that adding MediPicto pictograms was a good idea. Some even responded enthusiastically to the suggestion, saying that it would have helped.
- Emotions
  Remote monitoring gave rise to a range of unexpected emotional responses from patients, who reported feeling grateful, annoyed, afraid, demeaned, guilty, and discriminated against.

Two patients expressed gratitude for being included in the program. Patients were happy to create a connection with the care team. They felt that they made the care team happy by accepting the tool and answering correctly. They wanted to please the care team because it was the care team who was looking after them. Patients liked chatting with the nurse care coordinator via the messaging system.

Four patients said they worried about doing things wrong, answering incorrectly, or making spelling mistakes. Two patients checked their spelling before replying. One patient expressed a lack of confidence in the computer network.

Four patients were annoyed at having to use a computer and gave up. Password and connection problems recurred for all four of these patients, which one patient found irritating. The patients did not state their difficulties outright when filling in the questionnaire, but some expressed annoyance because they were unsure of their answers.

Two patients were self-critical, judging themselves and their skills in a negative light. They expected to be unable to learn to use this new tool. One patient reported that he had never been able to use digital tools and typed with one finger.

Two patients repeatedly expressed feelings of guilt about not responding and not knowing what to do. During the interviews, they seemed to imply that they had not filled in the questionnaires properly.

Two patients felt that they were being discriminated against. During the interview, all patients were asked whether they could read and write in French. Two patients found this discriminatory. They did not state this outright. However, one patient raised his voice and expressed his displeasure forcefully. The other patient abruptly changed his attitude and tone when the question was asked. During the rest of the interview, he only answered yes or no, and wanted the interview to end quickly. The patients were immediately informed that all patients were asked this question during the interview (Fig. 4).

The interviews showed that the tool generated negative emotions which could impact the level of patient adherence.

## Discussion

Among patients in digital divide, 45% of them used telemonitoring with good or very good compliance with the “ePRO program.” ePRO adhesion is a form of treatment adherence, which “is the adoption by a person of a behavior that provides potentially positive expected results, in accordance with the prescription or advice of a healthcare professional” [32]. No association with social deprivation was seen. The nurse navigator managed 84% of alerts by herself. However, most patients included were bad or very bad questionnaire completers, illustrating their continuing reluctance to use the ePRO application. Despite understanding the clinical benefit of telemonitoring, patients under-used Onco’nect® and experienced strong emotional responses while using it.

Our results are consistent with the literature. In their 2023 study, Natori et al. showed that cancer patients were less likely to answer questionnaires if they were aged 65 years or older, were of Hispanic or Latino ethnicity, were living without a partner, or were receiving no treatment [33]. Furthermore, patients in that study who did not complete remote monitoring questionnaires were more likely to be referred to ERs and hospitalized. In 2018, Anthony et al. analyzed the use of digital portals by 2325 patients. They showed that non-users of digital portals were mostly men who were aged over 65 years, had no college education, were receiving Medicaid, were living in a rural area, and preferred to speak directly to their provider [34].

In our study, the nurse navigator gauged the seriousness of each alert by telephoning the patient to assess their symptoms and determining the action to be taken based on her clinical analysis and expertise. If the AE was a CTCAE grade 1, the nurse navigator advised the patient herself; if the grade was 2 or more, she shared the case with the patient’s oncologist and referred the patient to a family doctor or an appropriate hospital department, including the ER. The nurse navigator managed 84% of alerts by herself, which is slightly higher than the data in the literature. In their 2016 study, Laccetti et al. analyzed the use of MyChart, a personal health records portal at a National Cancer Institute-designated comprehensive cancer center. They showed that nurses managed 77% of interactions on digital tools [35]. Similarly, in the CAPRI study, nurses resolved 77% of alerts on their own [12]. The use of PROs in the Onco’nect® application may lead to red alerts that actually correspond to grade 1 CTCAE.

Several contradictory elements emerged from the analysis, notably between the enthusiasm for the questionnaire and lack of completion, between the lack of criticism of the questionnaire and interest in pictograms, and between the level of difficulty expressed by patients with the healthcare professional versus with the patient-partner. According to the literature, patients in socially precarious situations have poor literacy. We hypothesized that poor literacy among our patients may explain the low rate of good or very good completers.

The patient-partner identified patients’ difficulties with using the Onco’nect® application and created a useful step-by-step guide. Nowadays, patients are encouraged to be independent and take ownership of their health, for instance by using remote monitoring and PROs to monitor their tolerance to treatment AEs. This may make patients feel guilty if he or she has not “done well.” Persiani et al. have highlighted the limits of patient empowerment and hyper-responsibility, which may not be desired by all patients [36]. In our study, the tablet was given to patients at the first or second treatment in the outpatient clinic. Patients in the digital divide who have just been diagnosed with cancer may not be able to process the trauma of diagnosis *and* get to grips with a new communication tool [37–39].

The strength of our study is that it is the first to investigate remote monitoring of PROs combined with an educational program dedicated to cancer facing the digital divide. Our results highlight the issue of health literacy, a concept that is little known among clinicians. The weakness of the study is its small sample size and single-center nature. The interviewees were perhaps not representative of the entire study population as regards the following data points: gender, EPICES score, performance status, and tumor location. The interviews were conducted by the nurse navigator who oversees Onco’nect®, a fact which may have introduced bias into our qualitative analysis. The fact that incomplete questionnaires could not be submitted to the care team is a limitation of our study, which might give an indication of those who tried but found it too difficult.

Given these results, improvements to Onco’nect® have been planned. MediPicto pictograms will be incorporated into the next version of the application. It might be worth rewording the questions to make them accessible to as many patients as possible, and to offer a multilingual version of Onco’nect® [40]. It will be important that any such future improvements include input from patients facing the digital divide and social deprivation. Health literacy scales do not seem to adequately capture patients’ health literacy [41]. This is because completing the scales requires good basic literacy skills anyway, and is besides impractical in routine clinical settings. It would be useful to devise a scale that detects patients’ health literacy problems more easily.

## Conclusion

Dedicated support programs help many patients avail of digital health applications. However, most of our cancer outpatients facing the digital divide remained poor users of remote monitoring. Poor health literacy may explain our results and should be addressed in further studies.

## Supplementary Information

Below is the link to the electronic supplementary material.

Supplementary file1 (DOCX 646 KB)

## Data Availability

No datasets were generated or analysed during the current study.

## Abbreviations

AP-HP: Assistance Publique-Hôpitaux de Paris
IRB: Institutional review board
IQR: Interquartile range
NN: Nurse navigator
PP: Patient-partner
RE: Research engineer
ePRO: Electronic patient-reported outcome

## Declarations

### Institutional approval

The trial protocol was approved by Scientific and Ethics local institutional review board Mondor (IRB 00011558). Institutional review board Mondor approved this qualitative and quantitative single-center retrospective study on February 23, 2023. The study did not meet French criteria to be deemed human subjects research. Only data that was strictly necessary to the aims of our study were collected and analyzed. This study was conducted in accordance with the Declaration of Helsinki and reported using the STROBE and COREQ checklists for the quantitative and qualitative parts of our study, respectively.
