# Supplementary material for: Bridging the digital divide for outpatients treated with anticancer chemotherapy: a retrospective quantitative and qualitative analysis of an adapted electronic Patient Reported Outcome program
Source: Support Care Cancer. 2025 Jan 30;33(2):130. doi: 10.1007/s00520-025-09171-9 (PMC11782441; doi:10.1007/s00520-025-09171-9)
Supplement: Supplementary file 1 — Supplementary file1 (DOCX 646 KB) [file 520_2025_9171_MOESM1_ESM.docx]

Supplemental Data

| **Supp Table 1. Onco’nect alerts according to patient answers to ePRO questionnaires.** | | | | |
| --- | --- | --- | --- | --- |
|  | **Question** | **Answer** | **Orange alert** | **Red alert** |
| **1** | What is your temperature today or yesterday? | < 36°C |  | X |
|  |  | > 39°C |  | X |
| **2** | Over the last 7 days, how severe has your cough been at its worst? | No |  |  |
|  |  | Slight |  |  |
|  |  | Moderate |  |  |
|  |  | Severe |  | X |
|  |  | Very severe |  | x |
| **3** | In the last 7 days, how severe were your problems with tasting food or drinks at their worst? | No |  |  |
|  |  | Slight |  |  |
|  |  | Moderate |  |  |
|  |  | Severe | X |  |
|  |  | Very severe |  | X |
| **4** | In the last 7 days, how severe were your mouth ulcers (canker sores) or throat ulcers at their worst? | No |  |  |
|  |  | Slight |  |  |
|  |  | Moderate | X |  |
|  |  | Severe |  | X |
|  |  | Very severe |  | X |
| **5** | In the last 7 days, how much have mouth ulcers (canker sores) or throat ulcers interfered with your usual or daily activities? | Not at all |  |  |
|  |  | A little bit |  |  |
|  |  | Quite a bit |  |  |
|  |  | Very much | X |  |
|  |  | A great deal |  | X |
| **6** | In the last 7 days, how severe was the decrease in your appetite at its worst? | Aucune |  |  |
|  |  | Légère |  |  |
|  |  | Modérée |  |  |
|  |  | Sévère |  | x |
|  |  | Très sévère |  | x |
| **7** | Over the past 7 days, how has your decreased appetite affected your usual or daily activities? | Aucune |  |  |
|  |  | Légère |  |  |
|  |  | Modérée |  |  |
|  |  | Sévère | x |  |
|  |  | Très sévère |  | x |
| **8** | Over the past 7 days, how often have you experienced nausea? | Never |  |  |
|  |  | Rarely |  |  |
|  |  | Occasionally |  |  |
|  |  | Frequently |  | x |
|  |  | Almost constantly |  | x |
| **9** | In the last 7 days, how severe was your nausea at its worst? | Aucune |  |  |
|  |  | Légère |  |  |
|  |  | Modérée |  |  |
|  |  | Sévère |  | x |
|  |  | Très sévère |  | x |
| **10** | Over the past 7 days, how often have you experienced vomiting? | Never |  |  |
|  |  | Rarely |  |  |
|  |  | Occasionally |  |  |
|  |  | Frequently |  | x |
|  |  | Almost constantly |  | x |
| **11** | In the last 7 days, how severe was your vomiting at its worst? | No |  |  |
|  |  | Slight |  |  |
|  |  | Moderate | x |  |
|  |  | Severe |  | x |
|  |  | Very severe |  | x |
| **12** | Over the past 7 days, how often have you had loose or liquid stools (diarrhea)? | Never |  |  |
|  |  | Rarely |  |  |
|  |  | Occasionally |  |  |
|  |  | Frequently | x |  |
|  |  | Almost constantly |  | x |
| **13** | In the last 7 days, how severe was your shortness of breath at its worst? | No |  |  |
|  |  | Slight |  |  |
|  |  | Moderate | x |  |
|  |  | Severe |  | x |
|  |  | Very severe |  | x |
| **14** | In the last 7 days, how much has shortness of breath interfered with your usual or daily activities? | Not at all |  |  |
|  |  | A little bit |  |  |
|  |  | Quite a bit | x |  |
|  |  | Very much |  | x |
|  |  | A great deal |  | x |
| **15** | Have you had a rash in the last 7 days? | yes | x |  |
|  |  | no |  |  |
| **16** | Over the last 7 days, how severe has your hand-foot syndrome (rash on hands or feet that can cause chapping, peeling, redness or pain at its worst) been? | No |  |  |
|  |  | Slight |  |  |
|  |  | Moderate |  |  |
|  |  | Severe |  | x |
|  |  | Very severe |  | x |
| **17** | Over the past 7 days, how severe was the numbness or tingling in your hands or feet at its worst? | No |  |  |
|  |  | Slight |  |  |
|  |  | Moderate | x |  |
|  |  | Severe | x |  |
|  |  | Very severe | x |  |
| **18** | Over the past 7 days, how much has numbness or tingling in your hands or feet interfered with your usual or daily activities? | Not at all |  |  |
|  |  | A little bit |  |  |
|  |  | Quite a bit | x |  |
|  |  | Very much | x |  |
|  |  | A great deal | x |  |
| **19** | Over the past 7 days, how often have you felt pain? | Never |  |  |
|  |  | Rarely |  |  |
|  |  | Occasionally |  |  |
|  |  | Frequently | x |  |
|  |  | Almost constantly |  | x |
| **20** | In the last 7 days, how severe was your pain at its worst? | 0 |  |  |
|  |  | 1 |  |  |
|  |  | 2 |  |  |
|  |  | 3 |  |  |
|  |  | 4 |  |  |
|  |  | 5 |  |  |
|  |  | 6 |  |  |
|  |  | 7 |  | x |
|  |  | 8 |  | x |
|  |  | 9 |  | x |
|  |  | 10 |  | x |
| **21** | Over the past 7 days, how much has your pain interfered with your usual or daily activities? | Not at all |  |  |
|  |  | A little bit |  |  |
|  |  | Quite a bit |  |  |
|  |  | Very much | x |  |
|  |  | A great deal |  | x |
| **22** | Over the last 7 days, how severe has your fatigue, weariness or lack of energy been at its worst? | 0 |  |  |
|  |  | 1 |  |  |
|  |  | 2 |  |  |
|  |  | 3 |  |  |
|  |  | 4 |  |  |
|  |  | 5 |  |  |
|  |  | 6 |  |  |
|  |  | 7 | x |  |
|  |  | 8 | x |  |
|  |  | 9 | x |  |
|  |  | 10 | x |  |
| **23** | Over the past 7 days, how much has fatigue, lassitude or lack of energy interfered with your usual or daily activities? | Not at all |  |  |
|  |  | A little bit |  |  |
|  |  | Quite a bit |  |  |
|  |  | Very much | x |  |
|  |  | A great deal | x |  |
| **24** | In the last 7 days, how often did you feel that nothing could cheer you up? | Never |  |  |
|  |  | Rarely |  |  |
|  |  | Occasionally |  |  |
|  |  | Frequently | x |  |
|  |  | Almost constantly | x |  |
| **25** | Over the past 7 days, how severe has this feeling been at its worst? | No |  |  |
|  |  | Slight |  |  |
|  |  | Moderate |  |  |
|  |  | Severe | x |  |
|  |  | Very severe | x |  |
| **26** | Over the past 7 days, how much has this feeling interfered with your usual or daily activities? | Not at all |  |  |
|  |  | A little bit |  |  |
|  |  | Quite a bit |  |  |
|  |  | Very much | x |  |
|  |  | A great deal | x |  |
| **27** | Over the past 7 days, have you had a temperature above 38.3°C or below 36°C, or chills? | Oui | x |  |
|  |  | Non |  |  |
| **28** | Have you had any other symptoms you'd like to report? | Yes | x |  |
|  |  | No |  |  |
| **29** | What is/are this/these symptom(s)? | Free text |  |  |
| **30** | In the last 7 days, how severe was this symptom at its worst? | No |  |  |
|  |  | Slight |  |  |
|  |  | Moderate | x |  |
|  |  | Severe |  | x |
|  |  | Very severe |  | x |
| **31** | Have you had any new symptoms in the last 7 days for which you would like to be contacted? | Yes |  | x |
|  |  | No |  |  |
| **32** | Are you ready for your next chemo treatment? | 0 | x |  |
|  |  | 1 | x |  |
|  |  | 2 | x |  |
|  |  | 3 | x |  |
|  |  | 4 | x |  |
|  |  | 5 | x |  |
|  |  | 6 | x |  |
|  |  | 7 |  |  |
|  |  | 8 |  |  |
|  |  | 9 |  |  |
|  |  | 10 |  |  |
| **33** | Have you been in contact within 3 days with a person suffering from Covid 19? | Yes |  | x |
|  |  | No |  |  |
|  |  | I don't know |  |  |

Supplementary Table 2. Patient verbatim related to the overarching and subthemes summarizing patient issues when using Onco’nect.

| **Understanding of the clinical relevance of remote monitoring** | Benefits for patient care | *"It's to find out how I'm doing."* |
| --- | --- | --- |
|  |  | *"For good care... to better help us to better accompany us."* |
|  | Sense of security | *"Feeling how more secure."* |
|  |  | *"And then when I need them, they're there."* |
|  | Misunderstanding | *"Connecting with other patients too."* |
|  |  | *"It was offered to me, so I accepted it."* |
| **Poor level of health literacy** | Understanding the questionnaire    Daily reading | *"It may have a word or two that I can't understand."*  *"I used to buy newspapers, so I read."* |
|  |  | *"Read (pff) not so much no."* |
|  |  | *"To be honest, I don't read much."* |
|  | Interest in pictograms | *"It would be nice to have images like that."* |
|  |  | *"Why not the image with it can still help."* |
|  |  | *"It can still help sometimes that sometimes sentences we can't understand."* |
| **Emotional burden** | Fear | *"Were you afraid you'd misunderstood the question? Patient: Voilà!"* |
|  |  | *"Sometimes I make spelling mistakes.”* |
|  |  | *"I was making mistakes so, I was looking on the phone to see if it's the right one, how to see if it's well written."*  *"Fear of bad ticking."* |
|  | Devaluation | *"As little as I understand."* |
|  |  | *"I'm a little more evolved, though, huh."* |
|  |  | *«It's a block maybe like I don't master I don't know."* |
|  | Annoyance | *"That's what really gets me."* |
|  |  | *"The connection problems, did that get you into trouble? Patient: Yeah exactly."* |
|  | Discrimination | *"Oh I was born under the French flag, huh. Yes, I'm Algerian by birth, but..."* |
|  | Guilt | *"I tried to make it up to you."* |
|  |  | *"I confess."* |
|  |  | *"They're very, very nice people, aren't they? You have to be, in both senses."* |


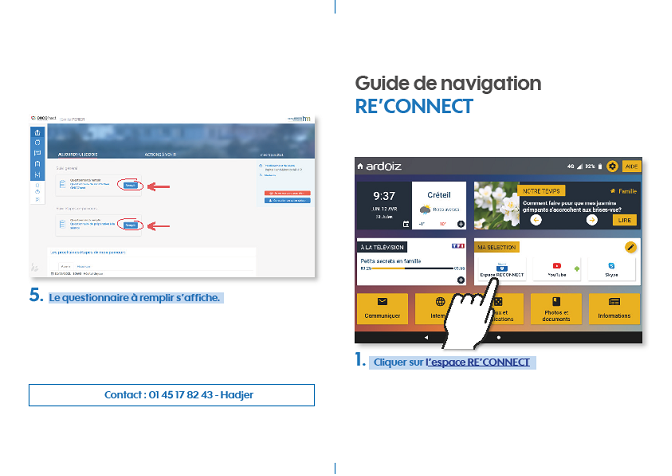
 
Supplementary Figure 1. Educational tools created by the patient partner

Cough
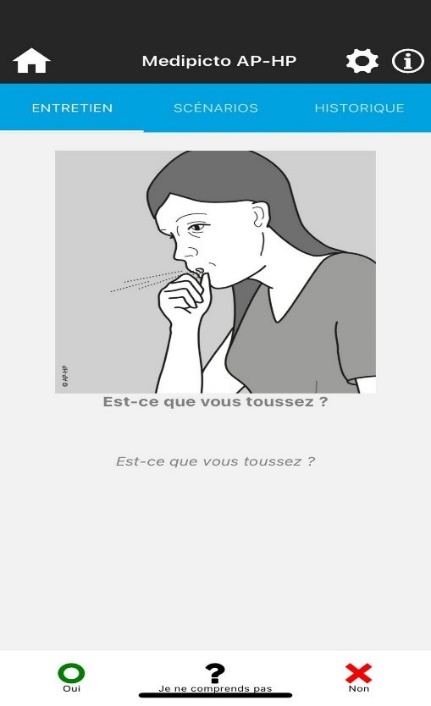
 Dysgeusia
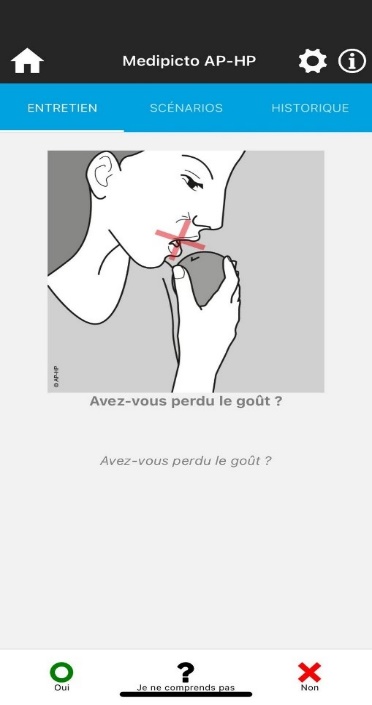


Anorexia
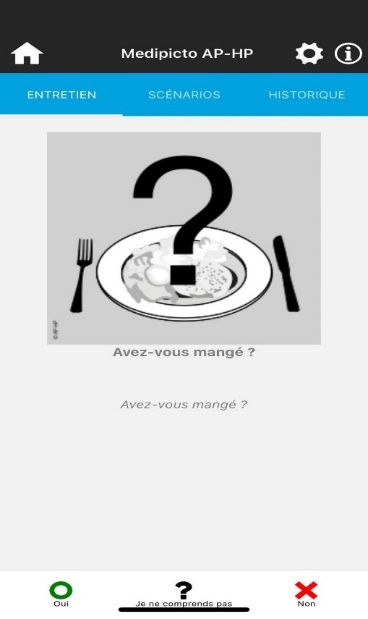
 Nausea
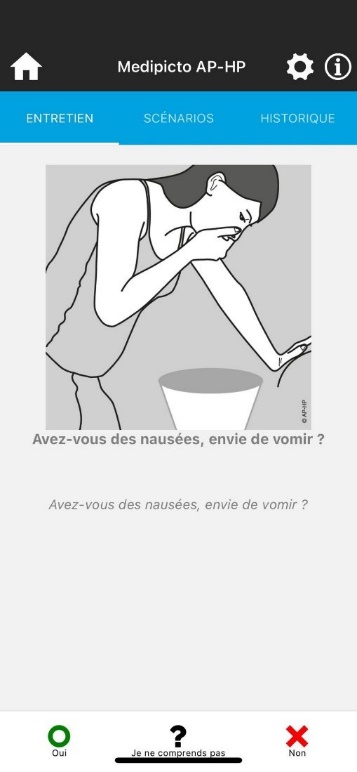


Vomiting  
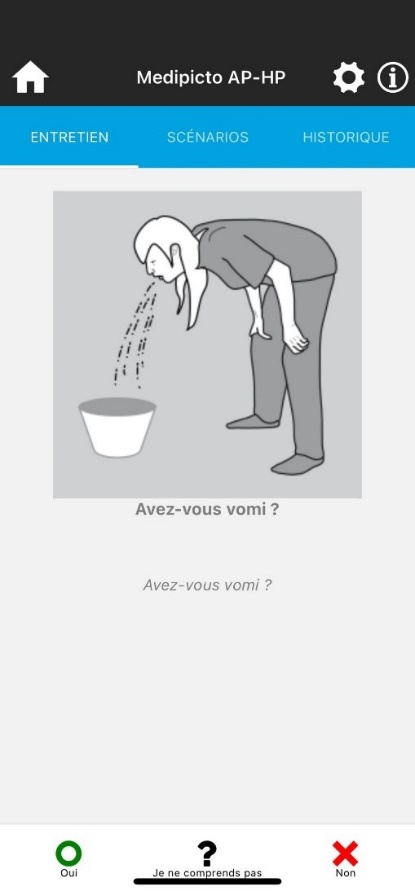
 Diarrhea
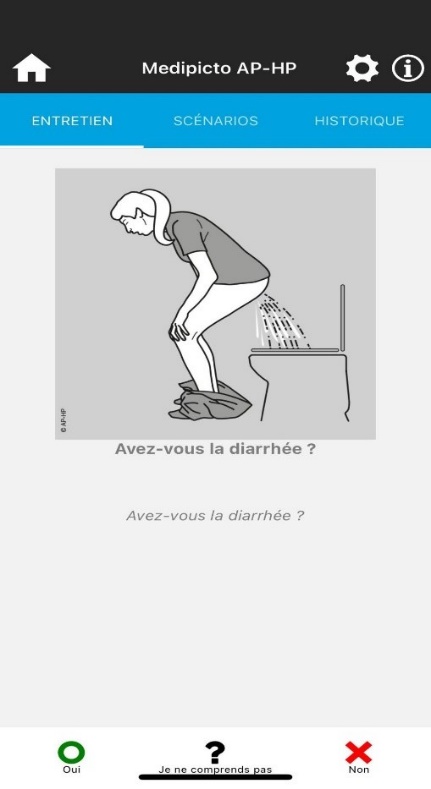


Dyspnea
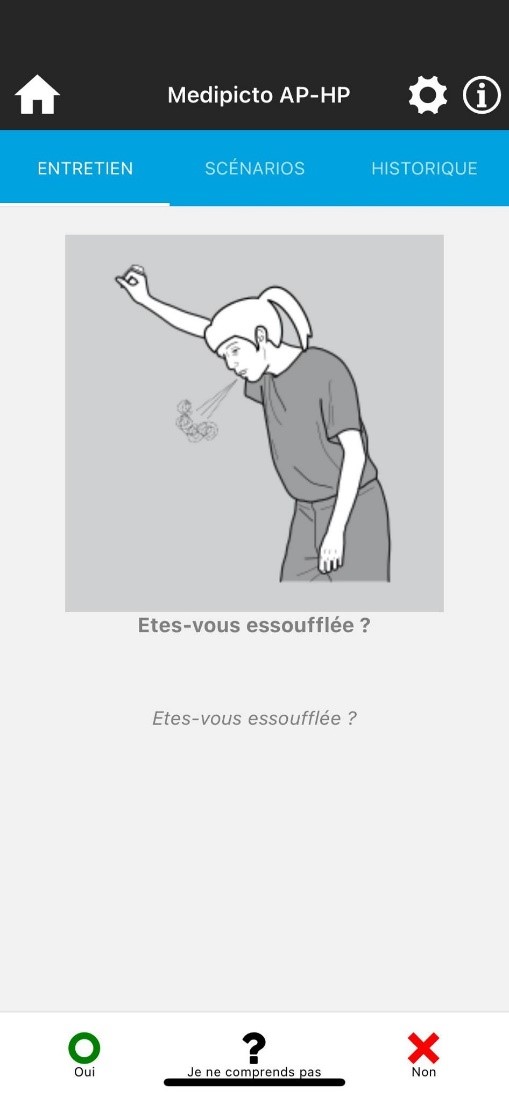
  Skin rash
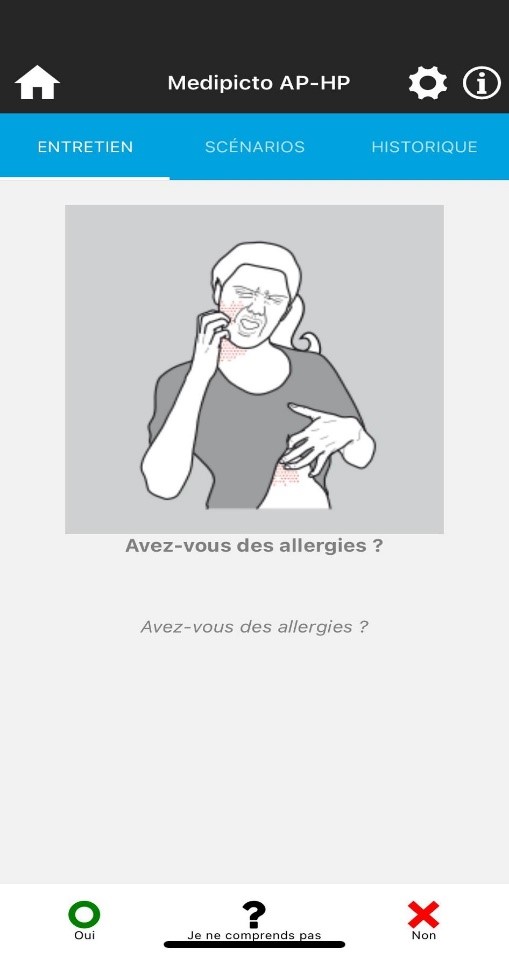


Pain
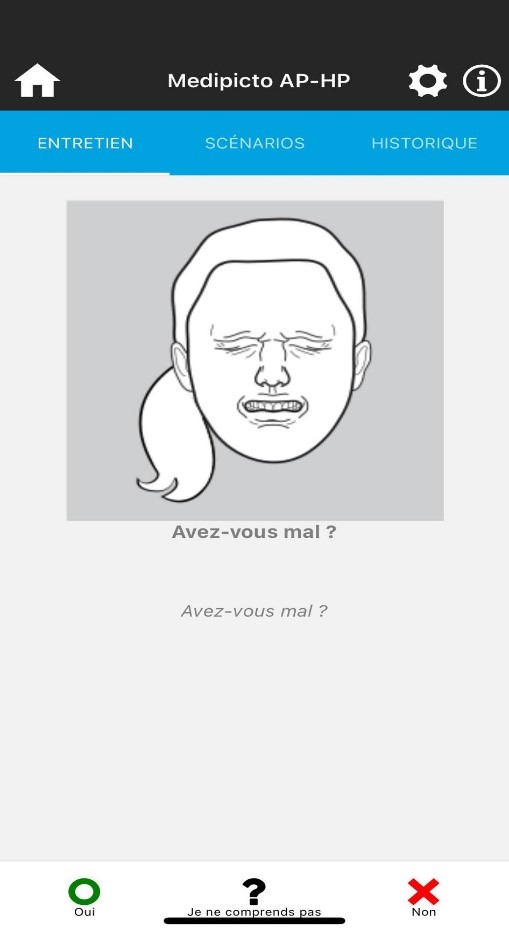
  Fatigue
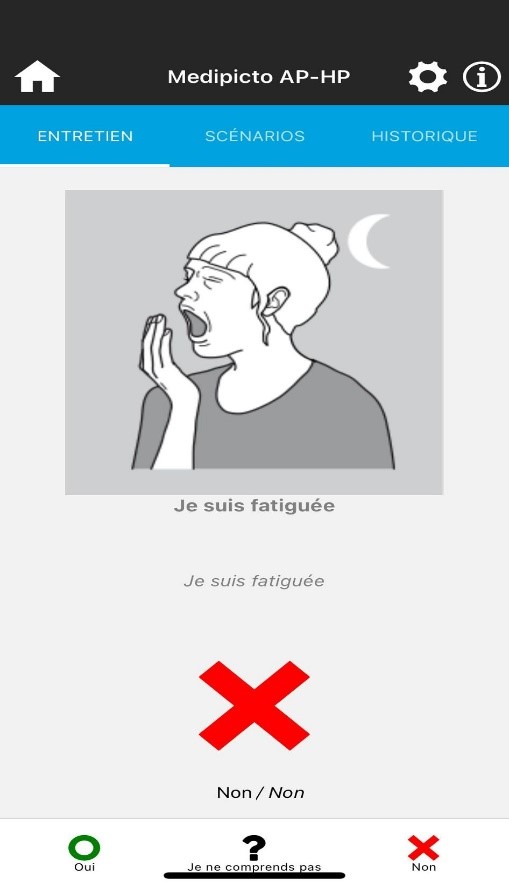


Fever
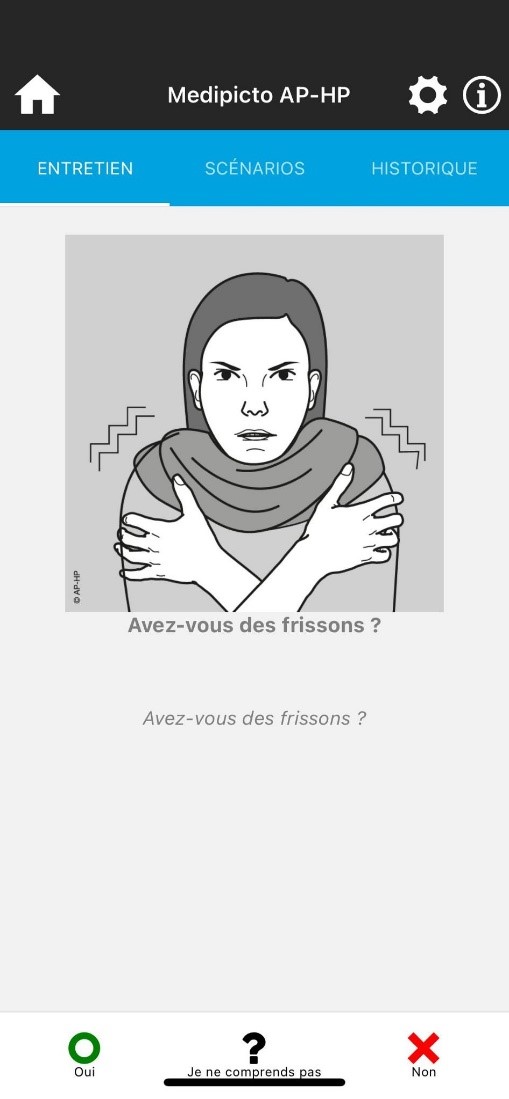

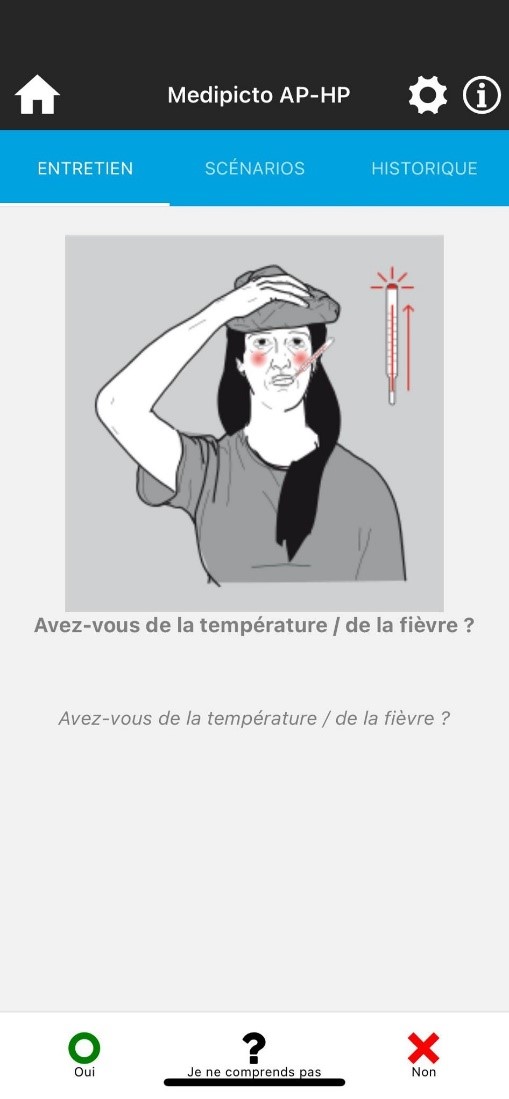


 Supplementary Figure 2. Médipicto AP-HP pictograms related to ePRO
